# Highly efficient dandelion-like near-infrared light photoinitiator for free radical and thiol-ene photopolymerizations

**DOI:** 10.1038/s41467-019-11522-0

**Published:** 2019-08-08

**Authors:** Zhiquan Li, Xiucheng Zou, Feng Shi, Ren Liu, Yusuf Yagci

**Affiliations:** 1 Key Laboratory of Synthetic and Biological Colloids, Ministry of Education, School of Chemical and Material Engineering, Jiangnan University, 214122 Wuxi, Jiangsu China; 2 International Research Center for Photoresponsive Molecules and Materials, Jiangnan University, 214122 Wuxi, Jiangsu China; 3 School of Materials Science and Engineering, Shaanxi Normal University, 710119 Xi’an, Shaanxi China; 4 Department of Chemistry, Faculty of Science and Letters, Istanbul Technical University, Maslak, 34469 Istanbul, Turkey

**Keywords:** Photocatalysis, Nanocomposites, Polymer synthesis

## Abstract

Efficient photopolymerization activated by nonharmful near-infrared (NIR) light is important for various biological applications. Here we propose a NIR light free-radical photoinitiator (PI) fabricated by incorporating oxime-ester coumarin functionality on the surface of upconversion nanoparticles (UCNPs). The coumarin groups of PI absorb the light emitted from the UCNP core, whereas the oxime ester groups undergo cleavage to form radicals. Upon irradiation at 980 nm, the mobile radicals, formed in a manner similar to that of dandelion seed release, initiate both free-radical and thiol-ene photopolymerizations. The superior efficiency of dandelion-like PIs assisted photopolymerizations can be attributed to the reduction of energy loss and increased local PI concentration due to Förster resonance energy transfer process and confinement effect, respectively. Moreover, the proposed PI system can initiate polymerization under low-power NIR laser and reduces the thermal side effects. The possibility of its potential use in deep curing applications was also demonstrated.

## Abstract (web summary)

Photo-induced polymerizations are being utilized to design and fabricate macromolecular structures with spatial and temporal control. Here the authors use a near-infrared light free-radical photoinitiator consisting of oxime-ester coumarin functionalities on the surface of upconversion nanoparticles for free-radical and thiol-ene photopolymerizations.

## Introduction

Various modes of photo-induced polymerization are being extensively utilized to design and fabricate macromolecular structures, which can provide excellent spatial and temporal control, and efficient productivity^1^. Plenty of methodologies can be found in recent literature studies^2–10^ that report photo-sensitive polymerizations for surface modification, micro–nano structure construction, and cross-linking processes to fabricate desired materials and high-precision devices. Besides conventional photopolymerization techniques, some of the latest photosensitive systems also fulfill the requirements of click reaction^11,12^. By using suitably selected multifunctional click-components, the orthogonal chemistry can be successfully applied to click-based photopolymerization, resulting in the formation of uniform networks with additional features, such as oxygen insensitivity and low shrinkage^13,14^. Noteworthy, these desirable features cannot be achieved with standard systems. To date, some click-based photopolymerization systems have been reported^15–19^. However, most of them exclusively require ultraviolet (UV) or visible light to trigger the reaction. In fact, regardless of the polymerization mode applied, most of the polymerization processes are conducted in this wavelength range. However, the use of high-energy UV photons can be detrimental to a majority of organic, inorganic, and biological species, which are sensitive to UV irradiation, and lead to undesirable side reactions^20^. Therefore, it is highly desirable to develop photoinitiating systems which can be activated by adequately lower energy photons, such as nonharmful near-infrared (NIR) light with longer-distance control^21,22^. In particular, this wavelength regime is of critical importance for in vivo biological applications as tissue is optically transparent facilitating deeper overall light penetration^20^.

Successful introduction of NIR light into photo-induced polymerization requires appropriate photoinitiators (PIs)/photosensitizers with optimal absorption, which should match well with the emission of NIR light source for efficient photon absorption. In contrast to conventional UV/visible-light PIs, limited NIR-light PIs are available and most of these PIs have only tail absorption in the given spectral range. Typical NIR PIs include bacteriochlorophyll a^23^, polymethines^24^, dye-borate, and cyanine derivatives^25–27^. Evidently, inadequate photon absorption results in forming a limited number of active species to initiate polymerization. Two-photon excitation is an alternative approach to realize photopolymerization using femtosecond pulsed NIR laser. However, high-intensity pulsed lasers (normal intensity: >10^6^ W cm^−2^)^28^, combined with time-consuming spot-by-spot curing process, restricts the effective utilization of two-photon polymerization. Furthermore, two-photon absorption only occurs at the laser focus. The femtosecond laser is not sufficiently focused while passing through the tissue; therefore, this strategy cannot be implemented in deep tissue applications^20^.

The utilization of lanthanide-doped upconversion nanoparticles (UCNPs) is another promising approach based on photon upconversion of NIR light into UV or visible light to trigger various chemical processes. In recent years, UCNP-assisted reactions have garnered significant research attention because they can initiate polymerization^29^ under lower light intensity than typically required by two-photon absorption methods. Typical example of UCNP-assisted photochemical reaction involves the coupling of low molar mass compounds by nitrile imine-mediated tetrazole-ene cycloaddition^20^. Upon photolysis at 974 nm, rapid conversion of tetrazole into a reactive nitrile imine occurred, which was quantitatively converted into a pyrazoline cycloadduct with an electron-deficient double bond. The same approach has also been used in macromolecular systems^30–32^. Haupt et al.^33^ demonstrated that core–shell type cross-linked thin polymers can be produced on UCNPs by photopolymerization activated by visible or UV light emission from the NIR-excited UCNPs. The initiation of free radical and free-radical-promoted cationic photopolymerizations in solvent by using upconverting glass (UCG) has also been reported^34^. Fluorescein excited by light emitted from the UCG underwent electron-transfer reactions with pentamethyldiethylene triamine to form free radicals capable of initiating polymerization of methyl methacrylate (MMA). Compared to conventional irradiation sources, the free-radical-promoted cationic polymerization of cyclohexene oxide is more efficient. Recently, Boyer and Mia^35^ presented a straightforward method for visible light-mediated photopolymerization to form core-shell type structures with controlled architecture from the UCNPs surfaces. This approach require no additional PI because the photoactive chain transfer agent was attached on the surface of UCNPs. A similar strategy, based on NIR-induced reversible-deactivation radical polymerization (RDRP) was also applied by Ding et.al.^36^ using dithiocarbonyl compounds as the initiator–mediator combination and UCNPs as internal light sources. In this case, the initiators were contained in solution, and well-defined polymers were obtained. Our group also used UCNPs as internal light sources to accomplish photopolymerization in several resin systems for the preparation of thick materials with a maximum depth of 13.7 cm^29,37,38^.

Even though the UCNP-assisted photopolymerization requires only the upconversion photons transmitting the distance between a UCNP and a PI before being absorbed, the weak intensity of upconversion fluorescence (below 1% conversion efficiency of NIR light)^39^ significantly limits the radical-quantum yield, and consequently the efficiency of photopolymerization. Therefore, improvement of the efficiency of UCNP-assisted photopolymerization is essentially required for a wide range of applications. The photoinitiation rate is generally defined by the following Eq. (1)^40^:

1 $$R_{\mathrm{i}} = \frac{{2\,\phi \,\varepsilon \,f\,IC_{\mathrm{i}}}}{{N_{\mathrm{A}}h\nu }}$$

where *ϕ*, *ε*, *f*, *I*, *C*_i_, *N*_A_ and *hν* refer to the quantum yield of scission, molar absorption coefficient, PI efficiency (or the ratio of initiation events to radicals generated by photolysis), light intensity, PI concentration, Avagodro’s number, and photon energy, respectively.

Although the luminescence intensity of UCNPs can easily be increased by increasing the output power of NIR light, the thermal side effect may interfere with the applied system, particularly, in bioapplications where tissues may get destroyed^41^. Therefore, alternative strategies have been developed to improve the luminescence intensity of UCNPs. In particular, Singh and Sun^42,43^ encapsulated UCNPs with Au/Ag to create surface plasmon resonance between UCNPs and noble metals. However, the underlying mechanism was not clear and only a small improvement was achieved.

**Fig. 1:**
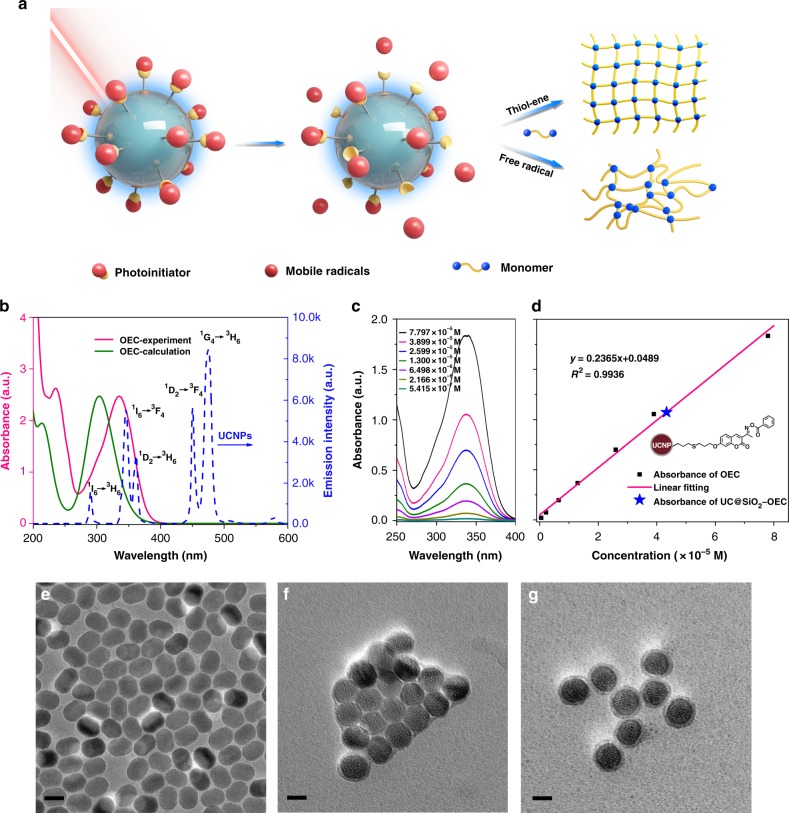
The structure characterizations of dandelion-like NIR light PIs. **a** The schematic illustration of photopolymerization by dandelion-like NIR light PIs. **b** The overlap of calculated and experimental UV–Vis absorption spectra of OEC, and the fluorescence emission spectrum of UCNPs. **c**, **d** UV–Vis absorption spectra of OEC at different concentrations and the related standard curve. **e**–**g** TEM images of UCNPs, UC@SiO_2_-SH, and UC@SiO_2_-OEC. Scale bar, 20 nm

As mentioned above, despite the feasibility of using UCNPs as NIR light absorber and a visible light emitter for photopolymerization processes, the existing methodologies render severe disadvantages. Herein, we report next-generation UCNPs by attaching the cleavable PIs on the surface of UCNPs, which act as dandelion-like PIs (Fig. 1a). In our design, augmentation of PI concentrations near the surface of UCNPs is expected to improve the efficiency of UCNP-assisted photopolymerization. Under the NIR irradiation, the incorporated PIs could absorb the emitted light from UCNPs to produce mobile active free radicals in a dandelion fashion to initiate the photopolymerization.

## Results

### Synthesis of dandelion-like near-infrared light photoinitiator

Endowed with their relatively high upconversion efficiency, *β*-NaYF_4_:18%Yb, 0.5%Tm nanoparticles were deliberately selected as the core and prepared by a solution-based method. The crystal structure and phase purity of the products are consistent with the previous results^29^. When excited under 980 nm laser irradiations, the UCNPs exhibited the following three groups of targeted emissions^44–46^: peaks centered at 451 and 474 nm corresponding to ^1^D_2_ → ^3^F_4_ and ^1^G_4_ → ^3^H_6_ transitions of Tm^3+^, respectively; peaks centered at ca. 345 and ca. 361 nm ascribed to ^1^I_6_ → ^3^F_4_ and ^1^D_2_ → ^3^H_6_, respectively; and a weaker emission peak centered at about 291 nm corresponding to the ^1^I_6_ → ^3^H_6_ transition of Tm^3+^ (Fig. 1b). For efficient photoinitiation, selection of suitable PIs is critical. Thus, the absorption spectrum of PIs should overlap with the emission spectrum of the UCNPs. Figure 1b presents that the calculated absorption spectrum of oxime-ester coumarin (OEC) matches well with the light emitted from UCNPs through ^1^I_6_ → ^3^H_6_, ^1^D_2_ → ^3^F_4_, and ^1^G_4_ → ^3^H_6_ transition of Tm^3+^ at 280–400 nm. The experimental absorption spectrum of OEC (centered at ca. 335 nm) even exhibited better overlap than the calculated one due to the solvation effect.

For the covalent attachment of PIs onto the surface of UCNPs, both PIs and UCNPs should possess antagonist reactive groups. However, the conventional synthesis of nanoparticles does not yield UCNPs with functional groups; and therefore, an additional modification is required. Thiol-ene click reaction appears to be the most suitable choice for the desired modification because of its high yield and selectivity. First, the surface modification of UCNPs was carried out by classic Stöber method^47^, wherein thiol group-functionalized silica was coated on the UCNPs. Then, the reactive PI component with a terminal double bond, as well as photosensitive coumarin and oxime ester groups, serving as chromophore and initiator, respectively, were designed (Supplementary Fig. 1). The subsequent thiol-ene reaction between the double-bond functionalized PI and UC@SiO_2_-SH yielded the desired dandelion-like UC@SiO_2_-OEC PIs (Supplementary Fig. 4)^48,49^. The morphology and size distribution of the intermediates (UC@SiO_2_-SH) and finally modified UCNPs (UC@SiO_2_-OEC) were investigated by transmission electron microscopy (TEM). Before modification, UCNPs were well patterned on the copper grid with uniform size of approximately 20 × 30 nm (Fig. 1e). After the silica coating, the hydrophilic surface of nanoparticles exhibited an aggregated trend and the size of the shell of UC@SiO_2_-SH was ~3–5 nm (Fig. 1f). Moreover, OEC conjugation did not alter the size of nanoparticles, but improved the dispersion due to the presence of hydrophobic OEC moiety on the surface (Fig. 1g). The reduced polydispersity further illustrates the nanoparticles become less aggregated (from 0.21 to 0.05, inset in Supplementary Fig. 5). The successful coating of silica with thiol groups and conjugation of OEC was also confirmed by fourier transform infrared (FT-IR) analysis (Supplementary Fig. 6). The FT-IR spectrum of UC@SiO_2_-SH exhibited the wide absorption bands in the range of 830 to 1280 cm^–1^, corresponding to the superimposition of various SiO_2_ peaks and Si–OH bonding. The absorption peaks at 1095 cm^–1^ (ν_as_[Si–O–Si]) and 670 cm^–1^ (ν_s_[Si–O–Si]), and the peak at ~2557 cm^–1^ (ν_s_[–SH]) in FT-IR spectrum of UC@SiO_2_-SH clearly confirm the presence of thiol group-functionalized silica coating on UCNPs (Supplementary Fig. 6). After the conjugation of OEC, the weakened thiol peak and newly emerged C–S peak at 960 cm^–1^ indicate the successful reaction of most of the SH groups with the double bonds in OEC.

Further evidence for the successful formation of UC@SiO_2_-OEC was obtained from the energy dispersive X-ray analysis (EDAX) (Supplementary Fig. 7). In addition to the peaks of the characteristic UCNPs elements, the EDAX spectrum of UC@SiO_2_-SH contains newly emerged peaks, corresponding to the Si and S elements, which indicates the presence of SiO_2_ shells and successful incorporation of SH groups. After conjugation of OEC by thiol-ene chemistry, the N signal is also detected in addition to the signals of the characteristic elements of UC@SiO_2_-SH, which confirms the successful conjugation of OEC moieties onto the surafce of UCNPs. Moreover, the uniform distribution of N element indicates the homogeneous dispersion of initiator molecules on the surface. Instead of covalent bonding, the possibility of physical adsorption of OEC on the surface of UCNPs was discarded by UV-vis spectral investigation. For this purpose, the solvent of UC@SiO_2_-OEC was sonicated, and the supernatant and nanoparticle precipitates were separately characterized by UV–vis spectral analysis (Supplementary Fig. 8). The supernatant solution did not exhibit any absorption at 335 nm, which remained the same with prolonged ultrasonication time and provided additional evidence for the successful preparation of the dandelion-like PI.

Supplementary Fig. 9 presents the TGA curves of UC@SiO_2_-SH and UC@SiO_2_-OEC, which indicate a thermal weight loss of ~6 and ~13 wt.% at 580 °C, respectively (Supplementary Table 1 and Notes). The observed difference in the weight loss corresponds to the mass fraction of the incorporated OEC (ca. 7 wt.%), which is close to the value obtained by UV–vis analysis. Notably, decomposition continued after 580 °C; however, did not reach the plateau value even at the high temperature of 800 °C. The enhanced thermal stability could be ascribed to the presence of dense silica coating, which influenced the degradation temperature of oleic acid on the surface of the UCNPs.

### Photoinitiation performance of dandelion-like photoinitiator

**Fig. 2:**
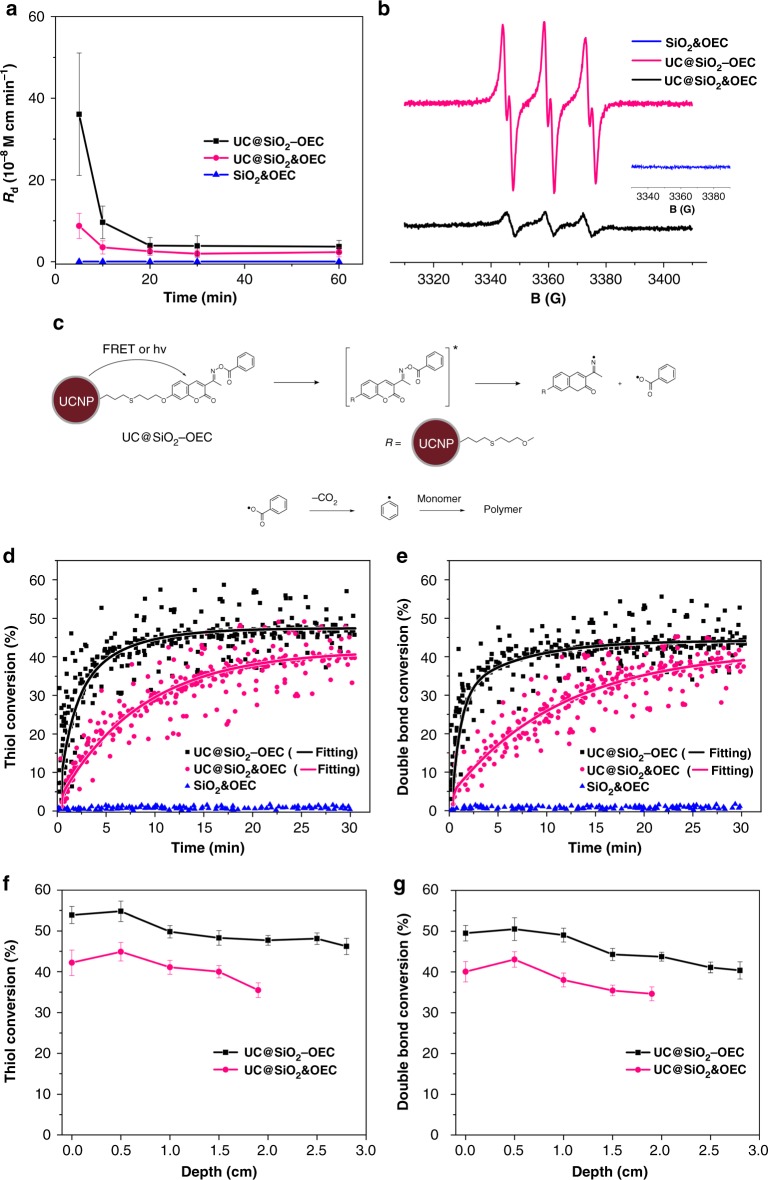
The photochemical properties of dandelion-like NIR light PIs. **a** The photolysis kinetics curves of UC@SiO_2_-OEC, UC@SiO_2_&OEC and SiO_2_&OEC. The error bars represent the standard error of the mean. **b** The electron spin resonance spectra of the radicals generated from UC@SiO_2_-OEC, UC@SiO_2_&OEC and SiO_2_&OEC and trapped by phenyl-*N*-tert-butyl-nitrone (PBN) in benzene. **c** The mechanism of photopolymerization by using UC@SiO_2_-OEC. The thiol-ene photopolymerization kinetics by using UC@SiO_2_-OEC, UC@SiO_2_&OEC and SiO_2_&OEC under 980 nm NIR laser. **d**, **e** The conversion vs. time profiles of thiol and double bond. **f**, **g** The conversion vs. depth profiles of thiol and double bond. The error bars represent the standard error of the mean

Furthermore, the photoinitiation performance of dandelion-like PI and its comparison with that of the physically mixed components were examined. First, the photodecomposition behavior of both systems was evaluated. Figure 2a and Supplementary Figs. 8c and 10b exhibit that the photodecomposition rate (*R*_d_) of UC@SiO_2_-OEC is 3.6 × 10^−7 ^M cm min^−1^, which is one order of magnitude higher than that of the mixed photoinitiation system, i.e., UC@SiO_2_ & OEC (*R*_d_ = 9 × 10^−8 ^M cm min^−1^). This is expected since the covalent attachments of the OEC enhanced the local initiator concentration on the surface due to the confinement effect. Therefore, under the identical irradiation conditions, UC@SiO_2_-OEC produced more free radicals than the mixed system, which was also confirmed by electron-spin-resonance (ESR) investigations. The ESR spectra exhibited only one spin adduct for both UC@SiO_2_-OEC/PBN and UC@SiO_2_&OEC/PBN systems when irradiated under 980 nm NIR light (Fig. 2b). The observed spin adduct corresponds to the known phenyl radical (*a*_N_ = 14.41 G, *a*_H_ = 2.21 G)^50^. However, the signal for UC@SiO_2_-OEC is much sharper and intense. Herein, it should also be pointed out that although the upconversion fluorescence is weaker than that of the conventional UV light, the covalent attachment of the PI can reduce the energy loss by decreasing the propagation distance of the upconversion fluorescence in the medium. Indeed, the spatial distance between OEC and UCNPs is less than 10 nm (Fig. 1g), which may result in non-radiative Förster resonance energy transfer (FRET) and further reduce the energy loss to boost radical-quantum yield. Supplementary Figs. 11 and 12 reveals that the emission spectrum of the UCNPs only decreases at the overlapping portion of the OEC absorption range rather than the entire region, which indicates the existence of FRET between UCNP and OEC moieties. FRET efficiency (*E*_FRET_) of UC@SiO_2_-OEC was calculated to be ca. 0.54, according to the Eq. (4). The time-resolved luminescence spectra further demonstrated the reduced lifetime of UC@SiO_2_-OEC compared with UC@SiO_2_&OEC, which indicated the emissions from UCNPs were reduced mainly by FRET in the UC@SiO_2_-OEC and radiation-reabsorption processes in the UC@SiO_2_&OEC, respectively (Supplementary Fig. 13). The results coincided well with the emissions of the fluorescence spectra. Therefore, UCNPs can effectively transfer energy to OECs to further generate free radicals to initiate photopolymerization. As the photolysis proceeds, the concentration of photoinitiating components gets reduced.

### Mechanism of the near-infrared photoinitiator

In order to eliminate the possibility of thermal impact of NIR light and nano effect on the process, pure silica nanoparticles with similar size distribution (inset in Supplementary Fig. 10d) were synthesized. Figure 2a, b and Supplementary Fig. 10d demonstrate that the system is composed of the mixture of silica nanoparticles and OECs cannot produce free radicals under applied NIR light irradiation conditions. Based on these results and previous studies, the probable mechanism for the radical generation process of dandelion-like NIR light PI is presented in Fig. 2c. The proposed mechanism indicates that, under the excitation of NIR light, the energy generated by the conversion of UCNPs is partially transmitted to the OEC moieties through a non-radiative form of FRET (*E*_FRET_ = 0.54), whereas the remaining energy is emitted and absorbed by OECs. The excited OECs, formed via both non-radiative FRET and absorption of the fluorescence emitting from UCNPs, or one of these two ways, undergo cleavage at the N–O bond, followed by a rapid release of CO_2_ to produce phenyl radicals. The irreversible decarboxylation step is particularly beneficial for the photopolymerization process because recombination of the initially formed radicals is significantly suppressed. Therefore, the amount of the excited state OECs in the first step is increased and the energy loss is minimized.

### Near-infrared-initiated radical polymerizations

Both types of NIR-initiated radical polymerizations, namely free radical and thiol-ene polymerizations using dandelion-like PI were studied to confirm the proposed mechanism and outline the scope of practical NIR curing systems (Table 1).

**Table 1:** UCNPs-assisted free radical and thiol-ene photopolymerizations with 980 nm NIR laser irradiation in DMF

Entry	PIs system^a^	Polym. mode^b^	Monomer	Excitation power [W cm^−2^]	*T* [min]	Conv.^c^ [%]	*M*_n_^d^ [KDa]
1	SiO_2_&OEC	Thiol-ene	BD-1/IADE	21.5	30	1.4	–
2	UC@SiO_2_&OEC	Thiol-ene	BD-1/IADE	21.5	30	27.8	21.0
3	UC@SiO_2_-OEC	Thiol-ene	BD-1/IADE	21.5	30	38.8	31.1
4^e^	UC@SiO_2_-OEC	Thiol-ene	BD-1/IADE	21.5	30	35.2	26.9
5	UC@SiO_2_-OEC	Thiol-ene	BD-1/IADE	21.5	240	54.3	32.7
6	UC@SiO_2_-OEC	Thiol-ene	BD-1/IADE	2.2	240	15.3	36.4
7	UC@SiO_2_&OEC	Free radical	MMA	16.8	30	14.7	17.2
8	UC@SiO_2_-OEC	Free radical	MMA	16.8	30	19.1	12.8
9	UC@SiO_2_-OEC	Free radical	MMA	16.8	240	28.4	14.3
10	UC@SiO2-OEC	Living radical	MMA	16.8	120	23.6	19.5
11	UC@SiO_2_-OEC	Free radical	*t*BA	16.8	30	20.3	13.9

^a^Identical concentration of photoinitiation component was controlled by UV-vis spectrum

^b^Monomers in thiol-ene and free radical photopolymerizations referred to butane-1,4-diyl bis(3-mercaptobutanoate) (BD-1)/3-bromopropene and isocyanuric acid diallyl ester (IADE) and methyl methacrylate (MMA) and *tert*-butyl acrylate (*t*BA), respectively

^c^Gravimetric determination of the conversion of the monomer

^d^Conversion of the monomer determined by gel permeation chromatography (GPC) according to polystyrene standards

^e^All the samples were degassed before irradiation except entry 4 to evaluate the effect of oxygen in thiol-ene system

Notably, the thiol-ene click photopolymerization was more efficient with UC@SiO_2_-OEC PI (Table 1, Entry 3) than with the mixed systems (Table 1, Entry 2) under similar reaction conditions. The kinetics of photopolymerization also rendered similar results. In order to evaluate the effect of oxygen in thiol-ene system, we conducted the UCNPs-assisted thiol-ene photopolymerizations with and without degassing (Table 1, Entries 3 and 4). The marginally changed conversion and final molecular weight indicate that the thiol-ene system has good oxygen tolerance. Higher conversion can be obtained via prolonging the irradiation time (Table 1, Entry 5). More importantly, despite the lower rate, the polymerization under reduced light intensity could still proceed using the as-described dandelion-like PI (Table 1, Entry 6), offering possibility for its application in bio-related systems. Utilization of the low-power NIR laser eliminates thermal side effects that cannot be achieved by regular UCNPs. Furthermore, applicability of the initiating system for conventional free radical polymerization was also studied. The UC@SiO_2_-OECs exhibited superior photoinitiation activity than the mixed systems in the NIR initiated polymerization of MMA (Table 1, Entries 7, 8 and 9). The potential application in controlled/living photopolymerization was also demonstrated in photo-RAFT polymerization of MMA yielding polymer with a narrow molecular weight distribution (Đ = 1.151) (Table 1, Entry 10, Supplementary Fig. 14 and Notes). The NIR photoinitiator was also versatile for other monomer, such as *t*BA (Table 1, Entry 11). In addition, the photopolymerization thiol-ene kinetics gave the same consequences for both rate, and thiol and double bond conversions (Fig. 2d, e). The enhanced activity in both polymerization types resulted from the high quantum yield and mobility of the radicals, which indicates that the proposed system can also be used for deep curing applications.

### Deep photopolymerization

Moreover, we also demonstrated that the curing depth of the thiol-ene system was increased by about 1 cm using the dandelion-like PI (Supplementary Fig. 15 and Notes). In the deep curing experiment, both thiol and double bond conversions were significantly higher than that by using the mixed systems (Fig. 2f, g). Furthermore, the cured material exhibited similar homogeneity, which is comparable to the previously reported conventional analogous^29,37^.

In summary, we designed a NIR dandelion-like PI by covalent bonding of a cleavable visible light sensitive OEC molecule on the surface of UCNPs. The results reveal that the covalent combination of OECs and UCNPs through functional SiO_2_ coating and thiol-ene click reaction is a strong and versatile strategy to achieve efficient NIR photopolymerization for a variety of applications. An important feature of the presented PI is the reduced energy loss and high local PI concentration due to FRET mechanism and confinement effect. Under the NIR irradiation, the mobile free radicals initiate both free radical and thiol-ene polymerizations more efficiently than the mixed photoinitiation systems. Furthermore, the enhanced efficiency was also demonstrated in deep-curing applications. Moreover, the initiation capability of the dandelion-like PI at low NIR light intensity overcomes the shortcomings associated with thermal influence using high power light sources. Therefore, the proposed PIs exhibit promise in bio applications.

## Methods

### Materials

Poly(oxyethylene)nonylphenyl ether (IGEPAL CO-520), tetraethoxysilane (TEOS), and 3-mercaptopropylmethyldimethoxysilane (MPTMS) were obtained from Sigma-Aldrich Chemistry, China. 2,4-Dihyoxybenzaldehyde, ethyl acetoacetate, and piperidine were purchased from Energy Chemical Co., Ltd. Phenyl-*N*-tert-butyl-nitrone (PBN), triallyl isocyanurate (TAIC), 3-bromopropene, cyanoisopropyl dithiobenzoate (CPDB) and isocyanuric acid diallyl ester (IADE) were purchased from Tokyo Chemical Industry, Shanghai. Butane-1,4-diyl bis(3-mercaptobutanoate) (BD-1) and 2,2-bis(((3-mercaptobutanoyl)oxy)methyl)propane-1,3-diyl bis(3-mercaptobutanoate) (PE-1) were supplied by Chembridge International Corp., methyl methacrylate (MMA), *tert*-butyl acrylate (*t*BA) and other reagents were supplied by Sinopharm Chemical Reagent Beijing Co., Ltd. Column chromatography was performed by conventional techniques on silica gel (200−300 mesh, Qingdao Haiyang Chemical Co., Ltd.), and silica gel plates were used for TLC analysis. MMA was purified using neutral alumina column to remove the inhibitor. The solvents were dried and purified by standard laboratory methods. All other chemicals were used without any further purification unless otherwise stated.

### Synthesis of allyl ether functional photoinitiator (OEC)

NaH (0.036 g, 1.5 mmol, 60%) and A4 (0.26 g, 1 mmol) were dissolved in dry tetrahydrofuran (10 mL) under N_2_ atmosphere. Then, the mixture was cooled to 0 °C and kept stirring for 20 min. Benzoyl chloride (0.15 mL, 1.3 mmol) was then added dropwise and the mixture was stirred for 20 min. The solution was quenched with aqueous sodium bicarbonate solution (5.0%, 10 mL) and extracted with dichloromethane (3 × 50 mL). Subsequently, the organic phase was dried over anhydrous sodium sulfate, filtered and evaporated under reduced pressure. The raw product was purified by column chromatography (petroleum ether/ethyl acetate = 5:1) to give pale yellow powder with a yield of 90%. ^1^H NMR (400 MHz, CDCl_3_) *δ* 8.18–8.13 (m, 2H), 7.66 (t, *J* = 7.4 Hz, 1H), 7.59-7.46 (m, 4H), 6.93 (dd, *J* = 8.6, 2.3 Hz, 1H), 6.87 (t, *J* = 4.1 Hz, 1H), 6.08 (ddd, *J* = 22.5, 10.6, 5.3 Hz, 1H), 5.48 (dd, *J* = 17.3, 1.3 Hz, 1H), 5.39 (dd, *J* = 10.5, 1.1 Hz, 1H), 4.65 (d, *J* = 5.3 Hz, 2H), 2.57 (s, 3H). ^13^C NMR (101 MHz, CDCl_3_): *δ* 158.89, 158.21, 158.11, 154.81, 151.63, 138.80, 128.79, 127.20, 125.39, 124.98, 124.10, 123.91, 115.00, 113.99, 109.13, 107.58, 96.68, 64.69, 11.21. HRMS (*m*/*z*): [M + Na]^+^ calcd. for C_21_H_17_NO_5_, 386.0999; found, 386.1165. The detailed synthesis procedures were given in the Supplementary Fig. 1 and Supplementary Methods. The structure properties of precursors and OEC were presented in Supplementary Figs. 2, 3 and Supplementary Notes.

### Synthesis of photointiator functionalized UCNPs (UC@SiO_2_-OEC)

OEC (15 mg, 0.04 mmol), dibenzoyl peroxide (12 mg, 0.05 mmol), and UC@SiO_2_-SH nanoparticle solution (10 mL of 1 mg/mL) in *N*, *N*-dimethylformamide (DMF) were mixed in a Schlenk flask and deoxygenated for 30 min. The mixture was stirred for 12 h under N_2_ atmosphere at 70 °C. NaYF_4_:Yb,Tm@SiO_2_-OEC nanoparticles (UC@SiO_2_-OEC) were centrifuged and washed with DMF twice to remove the excess OEC. The synthesis of other nanopatricles, UC@SiO_2_-SH, UC@SiO_2_ and pure SiO_2_, were given in the Supplementary Methods^48,51^.

### Oxime-ester coumarin incorporation degree

The UV standard curve of OEC was first built by the absorbance values of different concentration of OEC solution in DMF to determine the linear relationship between absorbance and concentration of OEC. Then, the absorbance of UC@SiO_2_-OEC dispersion (1 mg mL^−1^) in DMF was measured. OEC incorporation degree was calculated by using the following Eq. (2)^52^:

2 $${\mathrm{OEC}}\,{\mathrm{incorporation}}\,{\mathrm{degree}}\,\left( {{\mathrm{wt}}{\mathrm{.}}\% } \right) = \frac{{C_{\mathrm{A}}M}}{C}$$

where *C*_*A*_ is the concentration of OEC corresponding to absorbance of UC@SiO_2_-OEC dispersion estimated from the OEC standard curve (mol L^−1^), *M* is molecular weight of OEC (g mol^−1^), and *C* is the concentration of UC@SiO_2_-OEC dispersion (mg mL^−1^).

### Photolysis study

The photolysis study of different photoinitiation systems, namely OEC (8.2 × 10^−5^ M), UC@SiO_2_-OEC, UC@SiO_2_&OEC and SiO_2_&OEC (0.17 mg mL^−1^), was carried out in DMF at room temperature. For OEC, the solution was irradiated by LED light at 365 nm with light intensity of 100 mW cm^−2^. For nanoparticle based PIs, the identical concentration of similar sized particles as determined by TEM was subjected to NIR laser irradiation (20 W cm^−2^) at 980 nm. The identical concentration of photoinitiation components was also confirmed by UV–vis spectrum and incorporation degree (comparison below also based on these preconditions). The different photoinitiation systems were dispersed in a cuvette and irradiated using 980 nm NIR laser (20 W cm^−2^). For each system, the UV-vis absorption spectra were recorded at different irradiation times and the rates of decomposition were calculated by using Eq. (3) according to the differential form of Lambert–Beer law as follows^38^

3 $$R_{\mathrm{d}}\left( {{\mathrm{M}}\,{\mathrm{cm}}\,{\mathrm{min}}^{ - {\mathrm{1}}}} \right) =- \frac{1}{\varepsilon }\,\frac{{{\mathrm{d}}A}}{{{\mathrm{d}}t}}$$

where *R*_d_ is decomposition rate (M cm min^−1^), *ε* is molar extinction coefficient of OEC, and d*A* is the change of absorbance during the time of d*t*.

### Förster resonance energy transfer (FRET)

FRET further confirms the observed emission spectrum of the UCNPs. Supplementary Fig. 11, indicates that the emission spectrum of the UCNPs decreases only at the overlapping portion of the absorption spectrum of OEC, rather than the entire spectrum, which indicates that FRET occurs between UCNPs and OEC. The FRET efficiency (*E*_FRET_) was calculated by using the following Eq. (4)^53^:

4 $$E_{{\mathrm{FRET}}} = 1-\frac{{I_{{\mathrm{DA}}}}}{{I_{\mathrm{D}}}}$$

where *I*_DA_ and *I*_D_ correspond to intensity of UCNPs in presence of OEC and intensity of UCNPs only, respectively.

### Electron spin resonance experiments (ESR)

ESR experiments were carried out using an EMXplus10/12 X-band spectrometer, at 100 kHz, under magnetic field modulation. The power intensity was adjusted to 20 mW. The investigated photoinitiation systems (10 mg mL^−1^) and phenyl-*N*-tert-butyl-nitrone (PBN, 1 mg mL^−1^) were dissolved in benzene and deoxygenated with nitrogen for 10 min before irradiation. The radicals were generated through photolysis, at room temperature, when exposed to the 980 nm NIR laser (24 W cm^−2^) for 10 min. The ESR spectra simulations were performed by using the Bruker Xenon software.

### Photopolymerizations under the NIR laser

The photopolymerizations in solution were conducted in 100 mL Schlenk tubes. For thiol-ene photopolymerization, BD-1 (5.8 g, 19.7 mmol) and IADE (4.2 g, 20.1 mmol) were dissolved in anhydrous DMF (20 mL). Appropriate portions of different photoinitiation systems (10 mg mL^−1^) were added into above-mentioned solution. The reaction mixture was degassed using standard Schlenk line techniques by five freeze-pump-thaw cycles prior to irradiation. Then, the degassed solutions were stirred and irradiated from flank with NIR laser (980 nm, Changchun New Industries Optoelectronics Technology) under different pumping powers. At the end of given time, polymers were precipitated into excess methanol and then dried under reduced pressure. For free radical photopolymerization, MMA (10 g, 99.9 mmol) was dissolved in DMF (10 mL). The solutions of different PI systems were added and after the irradiation, polymers were precipitated as described for thiol-ene photopolymerization. For photo-RAFT polymerization, MMA (3.3 g, 33 mmol), CPDB (25 mg, 0.1 mmol) and UC@SiO_2_-OEC (10 mg mL^−1^) were dissolved in toluene (1 mL). The mixed solution was degassed using standard Schlenk line techniques and irradiated for 2 h. The polymer was precipitated by excess methanol after exposure time. The precipitation was obtained by centrifugation (8 min, 11,000 rpm) and dried at 50 °C. The conversion of all the monomers were calculated according to equation 5:34

5 $${\mathrm{Conversion}}\,\left( \% \right) = \frac{{m_{\mathrm{c}}}}{{m_0}} \times {\mathrm{100\% }}$$

where *m*_c_ and *m*_0_ correspond to weight of dried polymer precipitation and the initial monomer, respectively.

The photocurable formulations were prepared by mixing PE-1/TAIC (mol/mol = 3:4) and PI (10 wt.%) via ultrasonic vibration until the photoinitiation systems were evenly distributed within the polymer matrix. The storage and thermal stability of the formulation were studied (Supplementary Tables 2, 3 and Notes). A piece of the thin silicone membrane (thickness = 0.5 mm), with a hole (radius = 7 mm) in the middle, was attached on a KBr tablet, and the investigated resin was filled into the hole. Another KBr tablet was used to cover the hole for the subsequent tests. The polymerization experiments were carried out using a Nicolet 6700 FT-IR spectrometer (Thermo Fisher Scientific, 500–4000 cm^−1^ wavelength range, resolution 8 cm^−1^) to monitor the conversion of different groups as a function of exposure time. A NIR laser unit was used as an irradiation source and the light intensity was adjusted to 24 W cm^−2^ for the tests. The polymerization profiles were recorded during 30 min irradiation at room temperature. The well-distributed PIs in cured samples were monitored by EDAX as shown in Supplementary Fig. 16 and Notes. The polymerization kinetic studies were conducted by monitoring the disappearance of the double bond and thiol groups. For each sample, the curing process was repeated three times and the results were randomly collected. The conversion of different groups were calculated by using Eq. (6) as follows^15^:

6 $${\mathrm{Conversion}}\,\left( \% \right) = \left[ {1-\frac{{A_tA_{{\mathrm{rb}}}}}{{A_0A_{{\mathrm{ra}}}}}} \right] \times {\mathrm{100\% }}$$

where *A*_t_ corresponds to the area of the double bond and thiol absorptions at 1000–800 (or 1650–1600) cm^−1^ and 2570 cm^−1^, respectively at time *t*; and *A*_0_ represents the initial area of the peak, while *A*_rb_ and *A*_ra_ represent the absorbance intensities of C=O before and after curing, respectively.

### Theoretical calculation methods

The optimization of molecular structure (OEC) was performed at PM6 and M06-2X/6-311G* level (iso-value = 0.03). The excited state energy and UV absorption spectrum of OEC were calculated using M06-2X/def2-TZVP by Gaussian 09 B.01.

## Supplementary information

Supplementary Information

## Data Availability

All data generated or analyzed during this study are included in the published article and its Supplementary Information, and are available from the corresponding author on reasonable request.
