# Supplementary material for: Highly efficient dandelion-like near-infrared light photoinitiator for free radical and thiol-ene photopolymerizations
Source: Nat Commun. 2019 Aug 8;10:3560. doi: 10.1038/s41467-019-11522-0 (PMC6687813; doi:10.1038/s41467-019-11522-0)
Supplement: Supplementary file 1 — Supplementary Information [file 41467_2019_11522_MOESM1_ESM.pdf]

Supplementary Information

Highly Efficient Dandelion-Like Near-Infrared Light Photoinitiator for Free Radical and Thiol-ene Photopolymerizations

Zhiquan Li,[1,2] Xiucheng Zou,[1] Feng Shi,[3] Ren Liu[*,1,2] and Yusuf Yagci[*,2,4]

[1] Key Laboratory of Synthetic and Biological Colloids, Ministry of Education, School of Chemical and Material Engineering, Jiangnan University, 214122, Wuxi, Jiangsu, China.

[2] International Research Center for Photoresponsive Molecules and Materials, Jiangnan University, 214122, Wuxi, Jiangsu, China.

[3] School of Materials Science and Engineering, Shaanxi Normal University, 710119, Xi'an, Shaanxi, China.

[4] Department of Chemistry, Faculty of Science and Letters, Istanbul Technical University, 34469, Maslak, Istanbul, Turkey.

Corresponding authors: R.L. (email: liuren@jiangnan.edu.cn) or to Y.Y. (email: yusuf@itu.edu.tr)

Table of contents

Supplementary Figures 3

Supplementary Tables 8

Supplementary Methods 9

Supplementary Notes 11

Supplementary References 13

Supplementary Figures

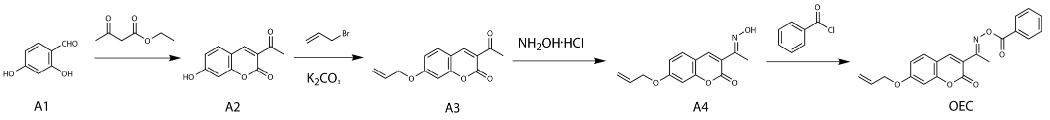

Supplementary Figure 1. The synthetic route of reactive oxime-ester coumarin photoinitiator (OEC).

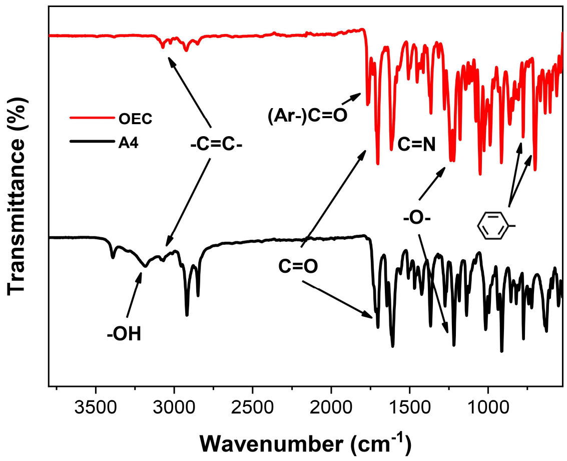

Supplementary Figure 2. FT-IR spectra of A4 and OEC.

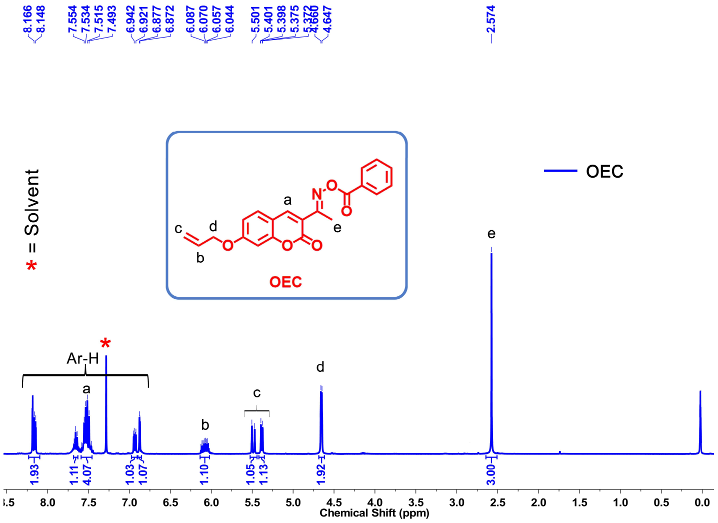

Supplementary Figure 3. ^{1}H-NMR spectrum of OEC.

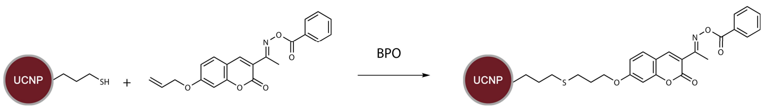

Supplementary Figure 4. The synthetic route of OEC functionalized upconversion nanoparticle, UC@SiO_2-OEC.

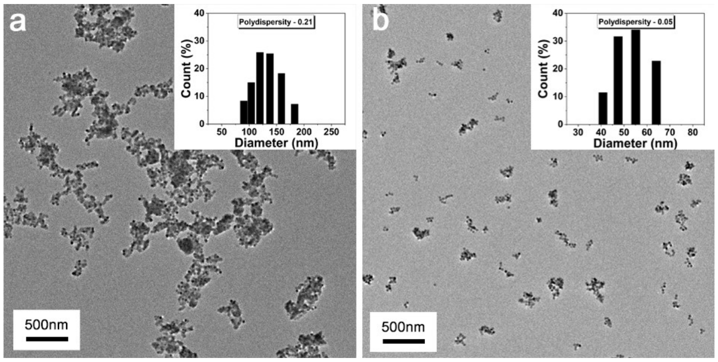

Supplementary Figure 5. The images of transmission electron microscope (TEM). **a** UC@SiO_2-SH. **b** UC@SiO_2-OEC. The inset is their corresponding particles size distribution histograms.

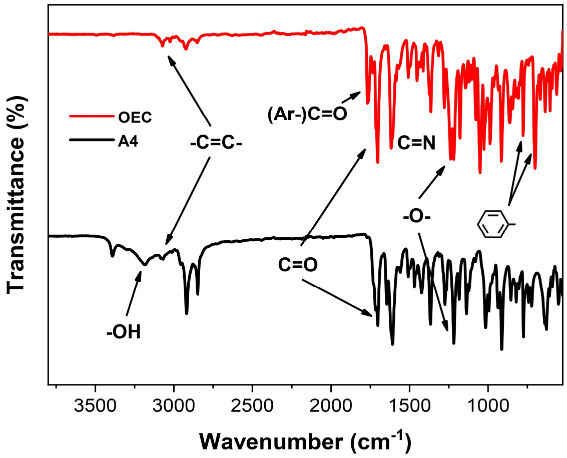

Supplementary Figure 6. FT-IR spectra of UCNPs、UC@SiO_2-SH and UC@SiO_2-OEC.

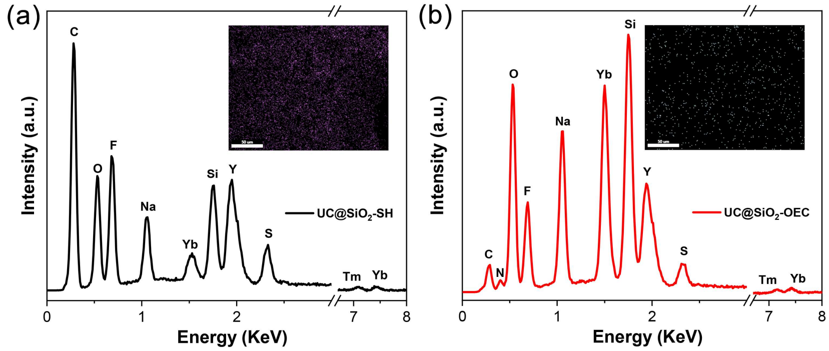

Supplementary Figure 7. Energy Dispersive Spectroscopy Analysis (EDAX). **a** EDAX spectrum of UC@SiO_2-SH and the image of S element distribution (inset). **b** EDAX spectrum of UC@SiO_2-OEC and the image of N element distribution (inset).

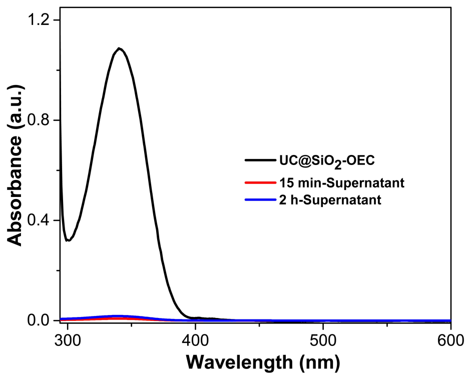

Supplementary Figure 8. UV-Vis absorption spectra of UC@SiO_2-OEC and the supernatant at different ultrasonic time.

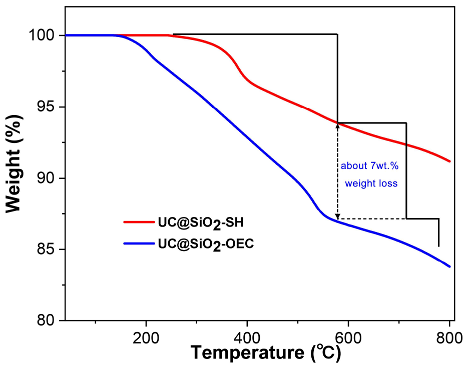

Supplementary Figure 9. Thermogravimetric analysis curve of UC@SiO_2-SH and UC@SiO_2-OEC.

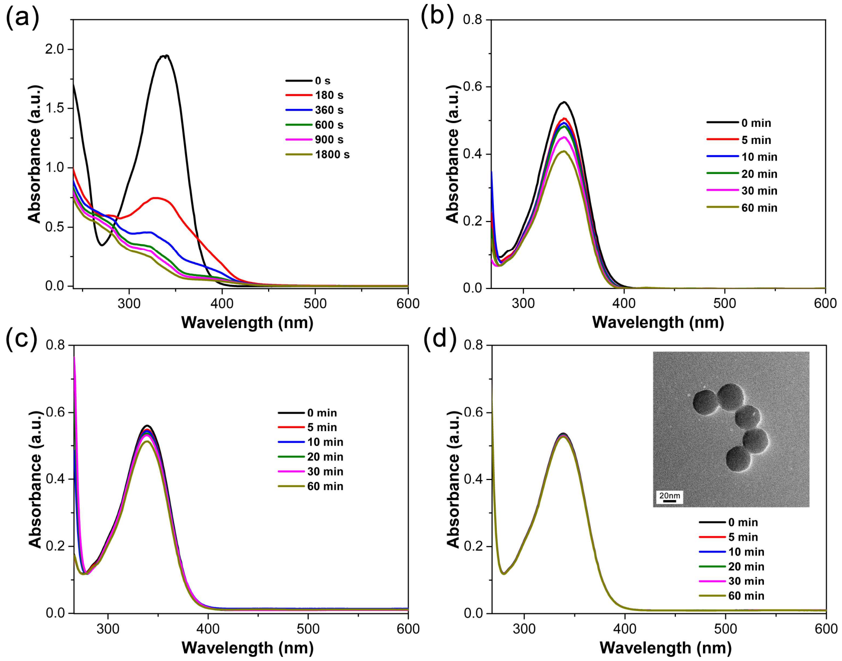

Supplementary Figure 10. UV-Vis absorption spectral changes of the photolysis. **a** The photolysis of OEC under the irradiation of 365 nm LED light. **b, c, d** The photolysis UC@SiO_2-OEC, UC@SiO_2&OEC and SiO_2 &OEC under the irradiation of 980 nm NIR laser. The inset in the photolysis of UC@SiO_2&OEC system is TEM image of synthetic SiO_2 microspheres.

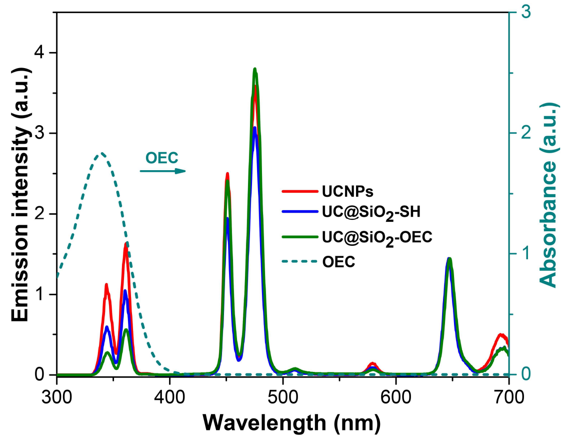

Supplementary Figure 11. The overlap of the fluorescence emission spectra of UCNPs, UC@SiO_2-SH and UC@SiO_2-OEC under 980 nm NIR laser and UV-Vis absorption spectrum of OEC.

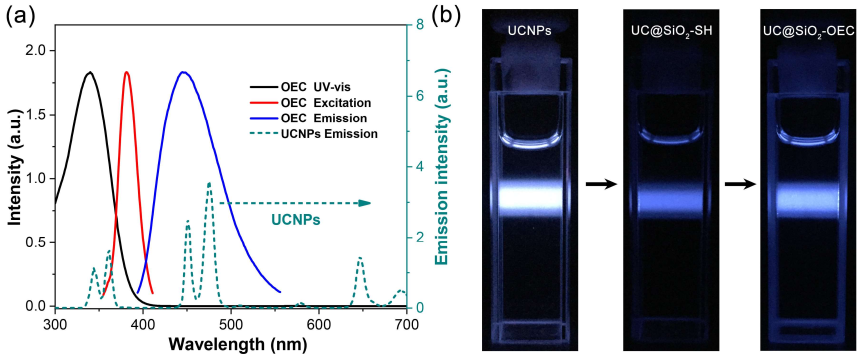

Supplementary Figure 12. The photophysical properties of OEC and PI. **a** The overlap of the UV-vis absorption spectrum, fluorescence excitation spectrum and fluorescence emission spectrum of OEC, and the fluorescence emission spectrum of UCNPs. **b** Fluorescence image of UCNPs, UC@SiO_2-SH and UC@SiO_2-OEC under 980 nm NIR laser.

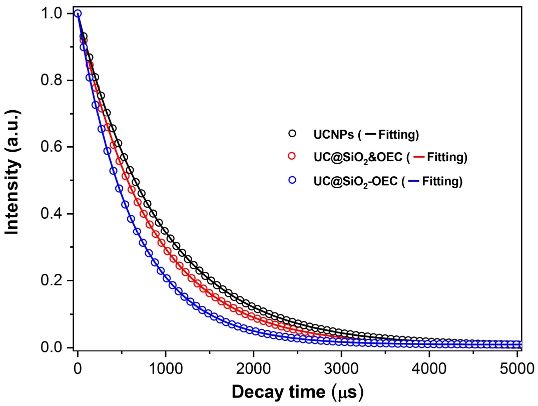

Supplementary Figure 13. The time-resolved luminescence spectra of UCNPs, UC@SiO_2&OEC and UC@SiO_2-OEC.

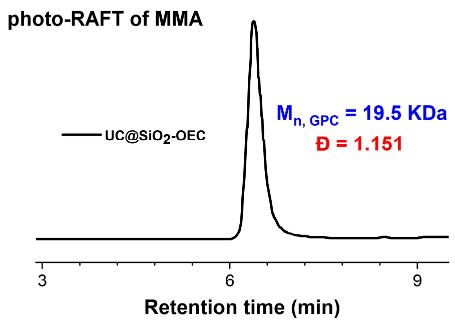

Supplementary Figure 14. The gel permeation chromatography (GPC) curve of PMMA by photo-RAFT polymerization with UC@SiO_2-OEC as photoinitiator.

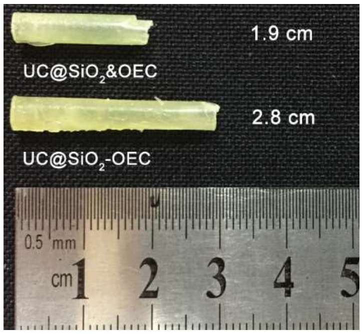

Supplementary Figure 15. The thiol-ene deep cured samples by using UC@SiO_2-OEC and UC@SiO_2&OEC as photoinitiation systems under 980 nm NIR laser.

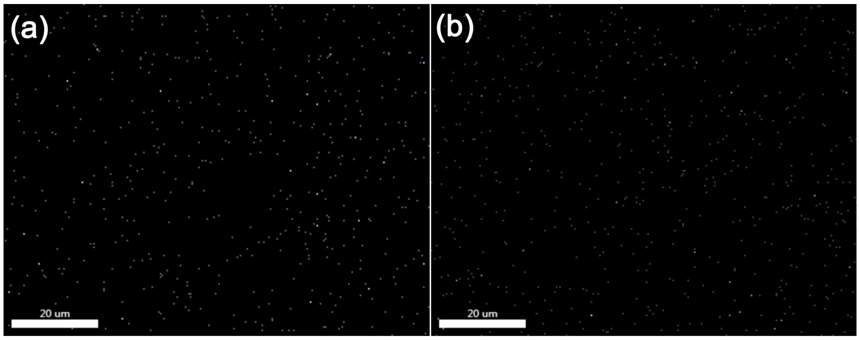

Supplementary Figure 16. The EDAX images of cured samples by PI. **a** N element distribution on the surface of cured samples. **b** N element distribution in cross-section of cured samples.

Supplementary Tables

Supplementary Table 1. The thermal weight losses of UC@SiO_2-SH and UC@SiO_2-OEC

Samples	Weight at 580°C (Loss) [%]
UC@SiO_2-SH	93.97 (6.03)
UC@SiO_2-OEC	87.04 (12.96)

Supplementary Table 2. The thermal gravimetric analysis of UC@SiO_2-OEC without monomers and the DSC measurements of acrylate and thiol-ene systems with and without UC@SiO_2-OEC

Samples	Decomposition temperature[a] [°C]	Onset temperature[b] [°C]
UC@SiO_2-OEC	199	-
TMPTA[c] (pure)	-	119 (132)
PE-1 / TAIC[c] (pure)	-	103 (116)

[a] Thermal gravimetric analysis for decomposition temperature of UC@SiO_2-OEC. [b] DSC measurements for onset temperature in formulations with and without UC@SiO_2-OEC. [c] The amount of new photoinitiator was added ~ 5 wt.%.

Supplementary Table 3. Viscosity values of different formulations after storage at different time

Samples	Viscosity[a] [mPa s]			
	0 day	1 day	3 day	7 day
TMPTA (pure)	85	85	85	85
TMPTA[b]	86	88	91	95
PE-1/TAIC (pure)	553	861	2977	31476
PE-1/TAIC[b]	557	1961	9387	89542

[a] Different formulations were storage at 60 °C and viscosity was measured at 25 °C. [b] The amount of new photoinitiator was added ~ 5 wt.%.

Supplementary Methods

^{1}H NMR (200 MHz) and ^{13}C NMR (50 MHz) measurements were carried out by a Bruker AVANCE III HD 400 MHz NMR spectrometer. The chemical shift (s = singlet, bs = broad singlet, d = doublet, t = triplet, and m = multiple) is stated in ppm using the nondeuterated solvent as the internal standard. The solvents with a grade of deuteration of at least 99.5% were used. High-resolution mass (HRMS) spectrometer measurements were performed using MALDI SYNAPT MS from Waters. Dynamic light scattering (DLS) measurements were carried out by ZetaPALS from Brookhaven. Viscosity tests were performed by MARS 60 from Thermo Fisher Scientific. UV−vis spectra were measured with a Beijing Purkinje TU-1901 UV−vis spectrophotometer. Fourier transform infrared spectra (FT-IR) were measured by using a Nicolet 6700 FT-IR spectrometer. Fluorescence excitation and emission spectra were measured by a CARY Eclipse fluorescence spectrometer. Time-resolved luminescence spectra were recorded using a chopped 978 nm laser as the excitation source from HORIBA Jobin Yvon Inc., Edison. Transmission electron microscope images were monitored with a JEOL JEM-2100 TEM and energy dispersive X-ray spectra were measured by a Hitachi S-4800 SEM with EDAX firmware. The thermal gravimetric analysis (TGA) experiments were performed by using Mettler Toledo TGA 1/1100SF with about 10 mg of the sample under a nitrogen atmosphere. The temperature was ramped from 25 to 800 °C at a heating rate of 20 °C min^{-1}. The differential scanning calorimetry (DSC) was carried out by using a PerkinElmer DSC-8000, with the sample of PIs dissolved in TMPTA. The solution concentration was ~ 5 wt.%, and the ramping rate was 10 °C min^{-1}.

Synthesis of hydroxyl functional ketocoumarin (A2): A2 was prepared according to the literature.[1]A mixture of 2,4-dihyoxybenzaldehyde (A1, 1.04 g, 10 mmol), ethyl acetoacetate (1.26 mL, 10 mmol) and piperidine (0.46 g, 5 mmol) was was refluxed in ethanol (20 ml) under N_2 atmosphere for 12 hr. After completion of the reaction, the solution was evaporated under reduced pressure and recrystallized with methanol. The raw product was purified by column chromatography (petroleum ether/ethyl acetate = 5:2) with a yield of 82%. ^{1}H NMR (400 MHz, $CDCl_3$) δ 7.66 (d, J = 9.5 Hz, 1H), 7.40 (d, J = 8.5 Hz, 1H), 6.94-6.81 (m, 2H), 6.28 (d, J = 9.5 Hz, 1H), 6.07 (ddd, J = 22.5, 10.6, 5.3 Hz, 1H), 5.42 (ddd, J = 13.9, 11.8, 1.3 Hz, 2H), 4.63 (dd, J = 4.0, 1.4 Hz, 2H).

Synthesis of allyl ether functional ketocoumarin (A3): A3 was synthesized in light of previous studies.[2] A2 (1.84 g, 9 mmol), 3-bromopropene (1.44 mL, 16.7 mmol) and anhydrous potassium carbonate (3 g, 21.7 mmol) was dissolved in 80 mL N,N-dimethylformamide. Then the solution was heated to 60 °C under N_2 atmosphere overnight. The solution was extracted with dichloromethane(3 × 80 mL) and washed by water(3 × 50 mL). Then the organic phase was dried over anhydrous sodium sulfate and the solvent was evaporated under reduced pressure. The raw product was purified by column chromatography (petroleum ether/ethyl acetate = 5:1) with a yield of 65%. ^{1}H NMR (400 MHz, $CDCl_3$) δ 8.54 (d, J = 18.3 Hz, 1H), 7.56 (t, J = 6.5 Hz, 1H), 6.94 (dd, J = 8.7, 2.4 Hz, 1H), 6.85 (dd, J = 9.0, 2.3 Hz, 1H), 6.16-5.93 (m, 1H), 5.57-5.28 (m, 2H), 4.74-4.58 (m, 2H), 2.73 (s, 3H).

Synthesis of allyl ether and hydroxylamine functional coumarin (A4): A mixture of A3 (0.24 g, 1 mmol), hydroxylamine hydrochloride (0.1 g, 1.5 mmol) and anhydrous sodium acetate (0.12 g, 1.5 mmol) were refluxed in ethanol (15 ml) under N_2 atmosphere for 4 h. Then the solution was cooled to room temperature and evaporated under reduced pressure. The raw product was purified by column chromatography (petroleum ether/ethyl acetate = 5:2) with a yield of 55%. ^{1}H NMR (400 MHz, DMSO) δ 11.34 (s, 1H), 8.04 (s, 1H), 7.72 (d, J = 8.6 Hz, 1H), 7.08-6.92 (m, 2H), 6.06 (ddd, J = 22.5, 10.6, 5.3 Hz, 1H), 5.42 (dt, J = 22.7, 11.4 Hz, 1H), 5.30 (dt, J = 17.8, 8.9 Hz, 1H), 4.70 (d, J = 5.3 Hz, 2H), 2.07 (s, 3H). ^{13}C NMR (101 MHz, DMSO) δ 162.03, 159.86, 155.65, 152.25, 141.51, 133.39, 130.55, 122.12, 118.64, 113.68, 112.90, 101.61, 69.38, 13.95. HRMS (m/z): $[M + Na]^{+}$ calcd. for $C_{14}H_{13}NO_4$, 282.0737; found, 282.0945. FT-IR spectrum of A4 was presented in Supplementary Figure 2.

Synthesis of allyl ether functional oxime-ester coumarin photoinitiator (OEC): NaH (0.036 g, 1.5 mmol, 60%) and A4 (0.26 g, 1 mmol) were dissolved in dry tetrahydrofuran (10 mL) under N_2 atmosphere. Then, the mixture was cooled to 0 °C and kept stirring for 20 min. Benzoyl chloride (0.15 mL, 1.3 mmol) was then added dropwise and the mixture was stirred for 20 min. The solution was quenched with aqueous sodium bicarbonate solution (5.0 %, 10 mL) and extracted with dichloromethane (3 × 50 mL). Subsequently, the organic phase was dried over anhydrous sodium sulfate, filtered and evaporated under reduced pressure. The raw product was purified by column chromatography (petroleum ether/ethyl acetate = 5:1) to give pale yellow powder with a yield of 90 %. ^{1}H NMR (400 MHz, $CDCl_3$) δ 8.18-8.13 (m, 2H), 7.66 (t, J = 7.4 Hz, 1H), 7.59-7.46 (m, 4H), 6.93 (dd, J = 8.6, 2.3 Hz, 1H), 6.87 (t, J = 4.1 Hz, 1H), 6.08 (ddd, J = 22.5, 10.6, 5.3 Hz, 1H), 5.48 (dd, J = 17.3, 1.3 Hz, 1H), 5.39 (dd, J = 10.5, 1.1 Hz, 1H), 4.65 (d, J = 5.3 Hz, 2H), 2.57 (s, 3H), as seen in Supplementary Figure 3. ^{13}C NMR (101 MHz, $CDCl_3$): δ 158.89, 158.21, 158.11, 154.81, 151.63, 138.80, 128.79, 127.20, 125.39, 124.98, 124.10, 123.91, 115.00, 113.99,

109.13, 107.58, 96.68, 64.69, 11.21. HRMS (m/z): $[M + Na]^+$ calcd. for $C_{21}H_{17}NO_5$, 386.0999; found, 386.1165. FT-IR spectrum of A4 was presented in Supplementary Figure 2.

Synthesis of silica-coated upconversion nanoparticle UC@SiO_2 and pure silica nanoparticle SiO_2: 120 µL of CO-520, 5 mL of cyclohexane and 5 mL of 4 mg/mL $NaYF_4$:Yb,Tm nanoparticle solution in cyclohexane were mixed and stirred for 10 min. 480 µL of CO-520 and 100 µL of 28 wt.% ammonia were then added and the container was sealed and sonicated for 20 min until a transparent emulsion was formed. 15 µL of TEOS was then added into the solution. The solution was stirred for 48 h at a speed of 220 rpm. $NaYF_4$:Yb,Tm@SiO_2 nanoparticles (UC@SiO_2) were precipitated by adding ethanol, and centrifuged and washed with ethanol/water (v/v=1:1) three times.[3]

5 mL of CO-520, 0.8 mL of 28 wt.% ammonia and 100 mL of cyclohexane were mixed and sonicated for 20 min until a transparent emulsion was formed. 1 mL of TEOS was then added into the solution and the solution was stirred for 48 h at a speed of 300 rpm. SiO_2 nanoparticles (SiO_2) were precipitated by adding ethanol, and centrifuged and washed with ethanol/water (v/v=1:1) three times.

Synthesis of thiol-functionalized upconversion nanoparticle UC@SiO_2-SH: 100 µL of CO-520, 5 mL of cyclohexane and 5 mL of 4 mg/mL $NaYF_4$:Yb,Tm nanoparticle solution in cyclohexane were mixed and stirred for 10 min. 400 µL of CO-520 and 80 µL of 28 wt.% ammonia were then added and the container was sealed and sonicated for 20 min until a transparent emulsion was formed. 10 µL of TEOS was then added into the solution. The solution was stirred for 24 h at a speed of 220 rpm. $NaYF_4$:Yb,Tm@SiO_2 nanoparticles were precipitated by adding ethanol, and washed with ethanol/water (v/v=1:1) three times and then re-dispersed in 5 mL cyclohexane. 1.0 mL of silica coated $NaYF_4$:Yb,Tm nanoparticles were added into 4 mL of 1.25 vol% MPTMS in cyclohexane, and stirred for 12 h at 20 °C with a speed of 300 rpm. The thiol-functionalized upconversion nanoparticles (UC@SiO_2-SH) were centrifuged and washed with ethanol/water (v/v = 1:1) twice to remove the excess MPTMS.[3]

Supplementary Notes

The structure of OEC functionalized upconversion nanoparticle (UC@SiO_2-OEC) by thermal thiol-ene reaction[4]: FT-IR analysis of the photoinitiator and its precursors is given in Supplementary Figure 6. The absorption peaks at 1095 cm^{-1} (ν_{as}[Si-O-Si]), and the peak around 2557 cm^{-1} (ν_s[-SH]) confirmed the thiol group-functionalized silica coating on UCNPs. After the conjugation of OEC, the weakened thiol peak and emerging C-S peak at 960 cm^{-1} indicated that the thiol-ene click chemistry was successful as most of the SH groups were consumed by reacting with double bonds in OEC. Energy dispersive X-ray analysis (EDAX) was also carried out to confirm the structure of UC@SiO_2-OEC (Supplementary Figure 7). In addition to the characteristic elements of UCNPs, the EDAX curve of UC@SiO_2-SH showed the appearance of characteristic elements, Si and S, indicating successful preparation of SiO_2 shells and introduction of -SH groups. After OEC was conjugated to UC@SiO_2-SH, the elements from UC@SiO_2-SH remained, and the N from OEC in UC@SiO_2-OEC can additionally be observed in the spectrum, manifesting the OEC conjugation onto the UCNPs surface.

To exclude the possibility of OEC adsorption on the surface of UCNPs instead of covalent bonding, the solvent of UC@SiO_2-OEC was sonicated, and the supernatant and nanoparticle precipitates were respectively characterized by UV−vis spectra analysis (Supplementary Figure 8).

OEC incorporation degree: TGA was used to analyze the incorporation degree of organic portion on the UCNPs surfaces (Supplementary Figure 9). The thermal weight losses of UC@SiO_2-SH and UC@SiO_2-OEC at 580°C were 6 wt.% and 13 wt.%, respectively (Supplementary Table 1). The difference of the weight loss correponds to the mass fraction of incorporated OEC (ca. 7 wt.%), which is close to the value obtained by UV analysis method.

Förster resonance energy transfer (FRET): The abnormal increase in the 400-500 nm range may be due to the fluorescence of OEC, which can be proved through fluorescence spectrum and digital photos (Supplementary Figure 12).

Reversible addition-fragmentation chain transfer photopolymerization (photo-RAFT) of MMA: Polymer with molecular weight and molecular weight distribution of ~19.5 KDa and Đ=1.151, respectively was obtained in photo-RAFT of MMA (Supplementary Figure 14).

Photopolymerizations under the NIR laser: For the deep curing application of photopolymerizations, the photocurable formulations were prepared by mixing PE-1/TAIC (mol/mol = 3:4) and UC@SiO_2-OEC (10 wt.%) via ultrasonic vibration until the photoinitiation systems were evenly distributed within the polymer matrix and no sedimentation was observed for 30 min before curing. Then, the photocurable samples were injected into a glass tube (5.05 cm long with outer radius of 5 mm and inner radius of 3 mm) which was then vertically exposed to a fiber coupled laser system for 20 min. The output power was adjusted to be 24 W cm^{-2} for the activation of the UCNPs. After irradiation, the cured samples were transferred out of the tube by breaking the glass and the uncured parts were removed by washing with acetone. The cured samples were vertically cut into a series of small parts with a length of 0.5 cm. The top layer (about 1 mm) of each parts were grounded into fine powders and then mixed with KBr powder (5 wt.%). A Fourier transform infrared spectrometer was used to measure the absorbance peaks of the uncured samples before irradiation and the cured samples (ATR-FTIR, Nicolet 6700, Thermo Fisher Scientific, 500–4000 cm^{-1} wavelength range, resolution 8 cm^{-1}, 6 scans per sample). The conversion of different groups was calculated by using equation 6.[5]

As shown in Supplementary Figure 15, the curing depth in PE-1/TAIC system using the dandelion-like photoinitiator increased by ~1 cm compared to the mixed system.

Dispersibility in cured sample: The well-distributed N element in energy dispersive X-ray spectra (EDAX) shows that the new PI can also be uniform in the cured sample because of N element only on the surface of UCNPs after photoinitiation (Supplementary Figure 16).

Stability tests: The thermal stability of UC@SiO_2-OEC was evaluated in the absence and in the presence of monomers by thermal gravimetric analysis and DSC measurement, respectively. As shown in Supplementary Table 2, the high decomposition temperature and relatively less reduced the onset temperatures, indicating that UC@SiO_2-OEC is thermally stable enough for daily storage.

The storage stability was examined by monitoring the viscosity of the formulation. The viscosity of the system is marginally changed for 7 days at 60 °C (Supplementary Table 3), indicating its excellent stability in the acrylic resin system. Inferior stability of the thiol-ene system was observed due to its high reactivity nature.

Supplementary References

1. Sepulveda, B., Quispe, C., Simirgiotis, M., Torres-Benítez, A., Reyes-Ortíz, J., Areche, C. & García-Beltrán, O. Gastroprotective activity of synthetic coumarins: Role of endogenous prostaglandins, nitric oxide, non-protein sulfhydryls and vanilloid receptors. *Bioorg. Med. Chem. Lett.* **23**, 5732-5735 (2016).
2. Mistry, S., Desai, S., Rao, S. & Shah, A. Synthesis of furocoumarins: Claisen rearrangement of 7-allyloxycoumarins. *Indian J. Heterocycl. Chem.* **4**, 301-306 (2004).
3. Bagheri, A., Arandiyan, H., Adnan, N., Boyer, C. & Lim, M. Controlled direct growth of polymer shell on upconversion nanoparticle surface via visible light regulated polymerization. *Macromolecules* **50**, 7137-7147 (2017).
4. Zhao. Y., Perrier. S. & Vana, P. Advances in Polymer Science: Controlled Radical Polymerization at and from Solid Surfaces Ch.3 (Springer Int. Publishing, Cham, 2016).
5. Li, Z., Zou, X., Zhu, G., Liu, X. & Liu, R. Coumarin-Based Oxime Esters: Photobleachable and Versatile Unimolecular Initiators for Acrylate and Thiol-Based Click Photopolymerization under Visible Light-Emitting Diode Light Irradiation. *ACS Appl. Mater. Interfaces* **10**, 16113–16123 (2018).
